# Structural Insight into the DNA-Binding Mode of the Primosomal Proteins PriA, PriB, and DnaT

**DOI:** 10.1155/2014/195162

**Published:** 2014-07-21

**Authors:** Yen-Hua Huang, Cheng-Yang Huang

**Affiliations:** ^1^School of Biomedical Sciences, Chung Shan Medical University, No. 110, Section 1, Chien-Kuo N. Road, Taichung City 40201, Taiwan; ^2^Department of Medical Research, Chung Shan Medical University Hospital, No. 110, Section 1, Chien-Kuo N. Road, Taichung City 40201, Taiwan

## Abstract

Replication restart primosome is a complex dynamic system that is essential for bacterial survival. This system uses various proteins to reinitiate chromosomal DNA replication to maintain genetic integrity after DNA damage. The replication restart primosome in *Escherichia coli* is composed of PriA helicase, PriB, PriC, DnaT, DnaC, DnaB helicase, and DnaG primase. The assembly of the protein complexes within the forked DNA responsible for reloading the replicative DnaB helicase anywhere on the chromosome for genome duplication requires the coordination of transient biomolecular interactions. Over the last decade, investigations on the structure and mechanism of these nucleoproteins have provided considerable insight into primosome assembly. In this review, we summarize and discuss our current knowledge and recent advances on the DNA-binding mode of the primosomal proteins PriA, PriB, and DnaT.

## 1. Introduction

**Figure 1:**
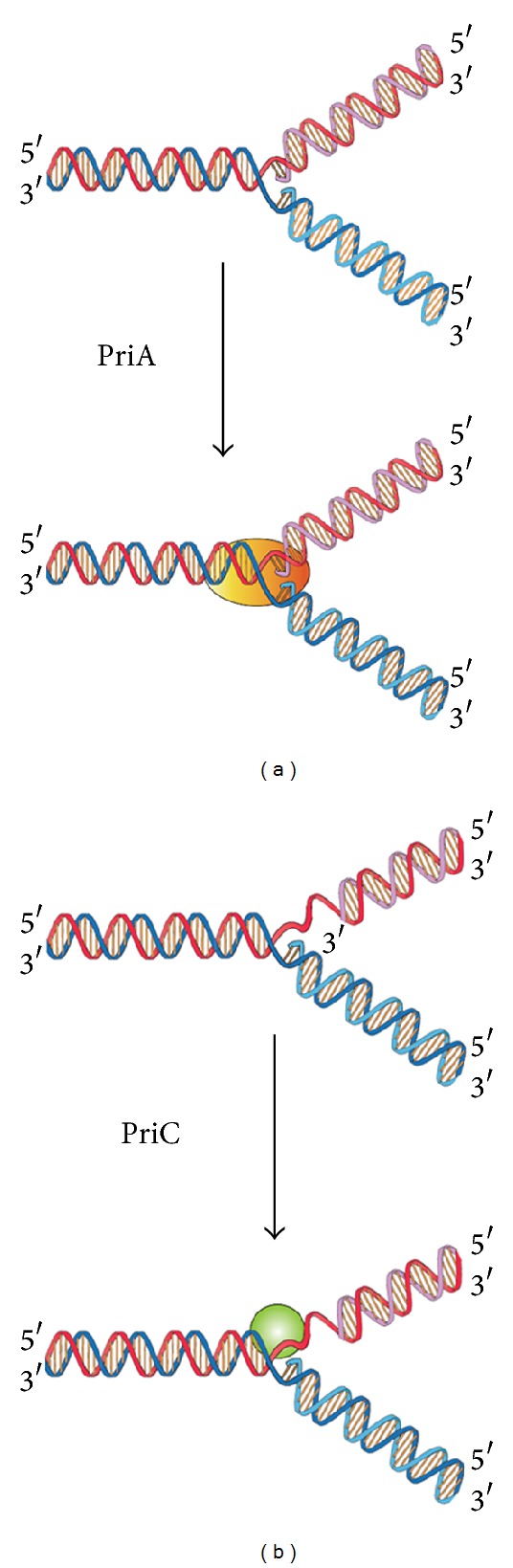
Two DnaB helicase-recruiting pathways for DNA replication restart at the stalled replication fork in vitro. The PriA-directed pathway (i.e., PriA-PriB-DnaT-DnaC-dependent reaction) preferentially uses fork structures without gaps in the leading strand, whereas the PriC-directed pathway (i.e., PriC-DnaC-dependent system) preferentially uses fork structures containing large gaps (>5 nucleotides) in the leading strand.

Genome integrity should be maintained from generation to generation to ensure proper cell function and survival [1–3]. In bacteria, some exogenous and endogenous sources of DNA damage can inactivate a large proportion of replication forks [4, 5]. When DNA is damaged, the replication machinery, originally initiated at* oriC*, can be arrested and disassembled anywhere along the DNA, leading to replication failure [5, 6]. To reload DnaB helicase for* oriC*-independent DNA replication, collapsed DNA replication forks must be reactivated by the replication restart primosome [7, 8]. Primosome is the protein complex responsible for the conversion of single-stranded circular DNA to the replicative-form DNA in the replication cycle of *ϕ*X174 phage [9, 10]. After DNA repair, the replication restart primosome [11–13], a formidable enzymatic machine, can translocate along the single-stranded DNA-binding protein (SSB), unwind the duplex DNA, and prime the Okazaki fragments required for the progression of replication forks [14]. In* Escherichia coli*, the replication restart primosome is composed of PriA helicase, PriB, PriC, DnaB helicase, DnaC, DnaT, and DnaG primase [3]. To date, two DnaB helicase-recruiting pathways are known: PriA-PriB-DnaT-DnaC-dependent and PriC-DnaC-dependent systems; the former system uses fork structures without gaps in the leading strand, whereas the latter system preferentially uses fork structures with large gaps (>5 nucleotides) in the leading strand [3]. As shown in Figure 1, PriA can bind directly and assemble a primosome on the template without gaps in the leading strand, and PriC initiates the assembly of a primosome on a fork containing gaps in the leading strand.

**Figure 2:**
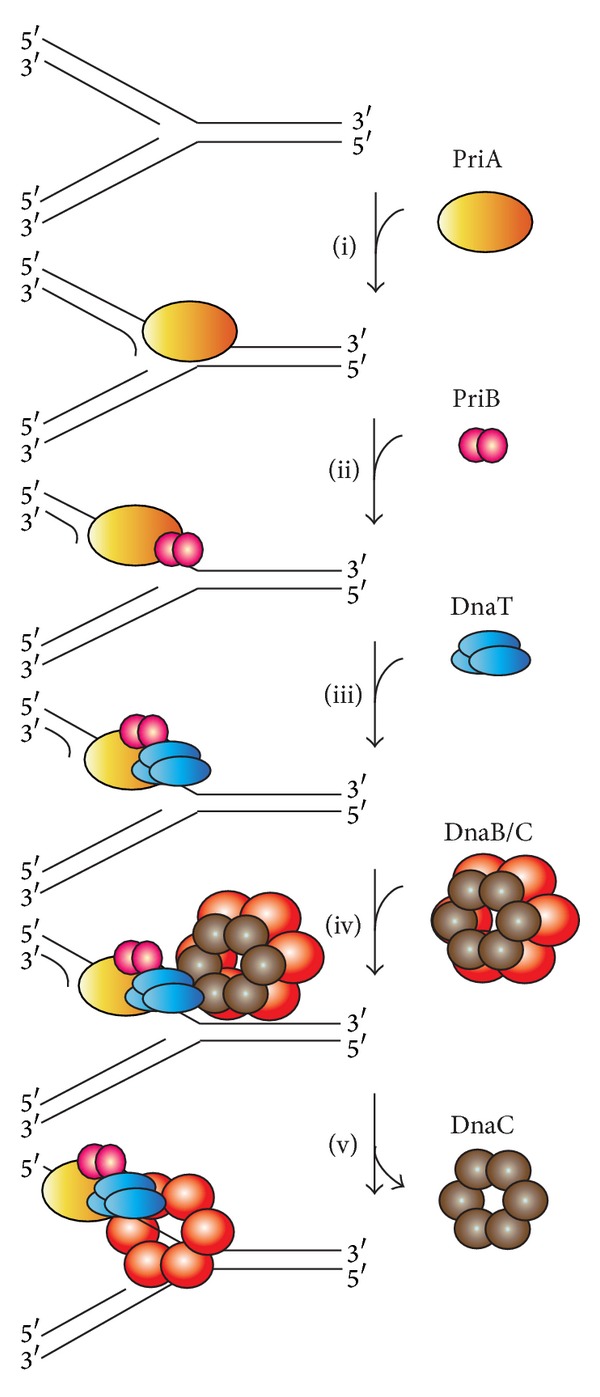
A hand-off mechanism for the replication restart primosome assembly. The proposed assembly mechanism is as follows. (i) PriA recognizes and binds to a replication fork, (ii) PriB joins PriA to form a PriA-PriB-DNA ternary complex, (iii) DnaT participates in this nucleocomplex to form a triprotein complex, in which PriB is released from ssDNA due to recruitment of DnaT, (iv) the PriA-PriB-DnaT-DNA quaternary complex loads the DnaB/C complex, and (v) DnaB is loaded on the lagging strand template.

A hand-off mechanism for PriA-directed primosome assembly [15] has been proposed (Figure 2), whereby (i) PriA recognizes and binds to a replication fork; (ii) PriB joins PriA to form a PriA-PriB-DNA ternary complex; (iii) DnaT participates in this nucleocomplex to form a triprotein complex, in which PriB is released from ssDNA due to recruitment of DnaT; (iv) the PriA-PriB-DnaT-DNA quaternary complex loads the DnaB/C complex; (v) DnaB is loaded on the lagging strand template. Genetic analyses suggest that these primosomal proteins are essential replication proteins for bacterial cell growth [12, 16–21]. These proteins are required for reinitiating chromosomal DNA replication in bacteria; thus, blocking their activities would be detrimental to bacterial survival [22, 23]. Several primosomal proteins, such as PriA, PriB, PriC, and DnaT, are not found in humans; thus, these proteins may be potential targets in developing antibiotics for the six antibiotic-resistant pathogens (*Enterococcus faecium*,* Staphylococcus aureus*,* Klebsiella pneumoniae*,* Acinetobacter baumannii*,* Pseudomonas aeruginosa*, and* Enterobacter* sp.) [24, 25]. The recently discovered inhibitor CGS 15943 targets* Neisseria gonorrhoeae* PriA helicase with an IC_50_ of 114 ± 24 *μ*M [26].

Over the past 10 years, considerable progress has been made in the structural mechanisms of the replication restart primosome assembly. The structural information is a prerequisite for formulating any model of the assembly mechanism of the primosome (Table 1). In the following sections, we summarize and discuss our current knowledge and recent advances on the DNA-binding mode of the primosomal proteins PriA, PriB, and DnaT.

## 2. Structural Insights into the DNA-Binding Mode

### 2.1. PriA Helicase

PriA functions as a scaffold that recruits other primosomal proteins. It was originally discovered as an essential factor for the conversion of single-stranded circular DNA to the replicative-form DNA of *ϕ*X174 single-stranded phage in vitro [27]. The* priA* mutant of* E. coli *exhibits complex phenotypes that include reduced viability, chronic induction of SOS response, rich media sensitivity, decreased homologous recombination, sensitivity to UV irradiation, defective double-stranded break repair, and both induced and constitutive stable DNA replication [6, 12, 28–30]. The native PriA is a monomer with a molecular mass of ~82 kDa. The tertiary structure of the monomer contains two functional domains, namely, the helicase domain (HD), which encompasses ~540 amino acid residues from the C-terminus, and the DNA-binding domain, which comprises ~181 amino acid residues from the N-terminus [31–33]. PriA is a DEXH-type helicase that unwinds DNA with a 3′ to 5′ polarity [34]. Fuelled by the binding and hydrolysis of ATP, PriA moves along the nucleic acid filaments with other primosomal proteins and separates double-stranded DNA into their complementary single strands [35]. PriA preferentially binds to a D-loop-like structure by recognizing a bend at the three-way branched DNA structures and duplex DNA with a protruding 3′ single strand [32, 36, 37]. PriA interacts with SSB [38], PriB [15, 39, 40], and DnaT [15]. PriA can unwind the nascent lagging strand DNA to create a suitable binding site to help PriC load the DnaB helicase onto stalled replication forks where a gap exists in the nascent leading strand [41, 42]. The crystal structures of the N-terminal 105 amino acid residue segment of* E. coli* PriA (EcPriA) in complex with different deoxydinucleotides show a feasible interaction model for the base-non-selective recognition of the 3′-terminus of DNA between the nucleobase and the DNA-binding sites of EcPriA [43].

**Figure 3:**
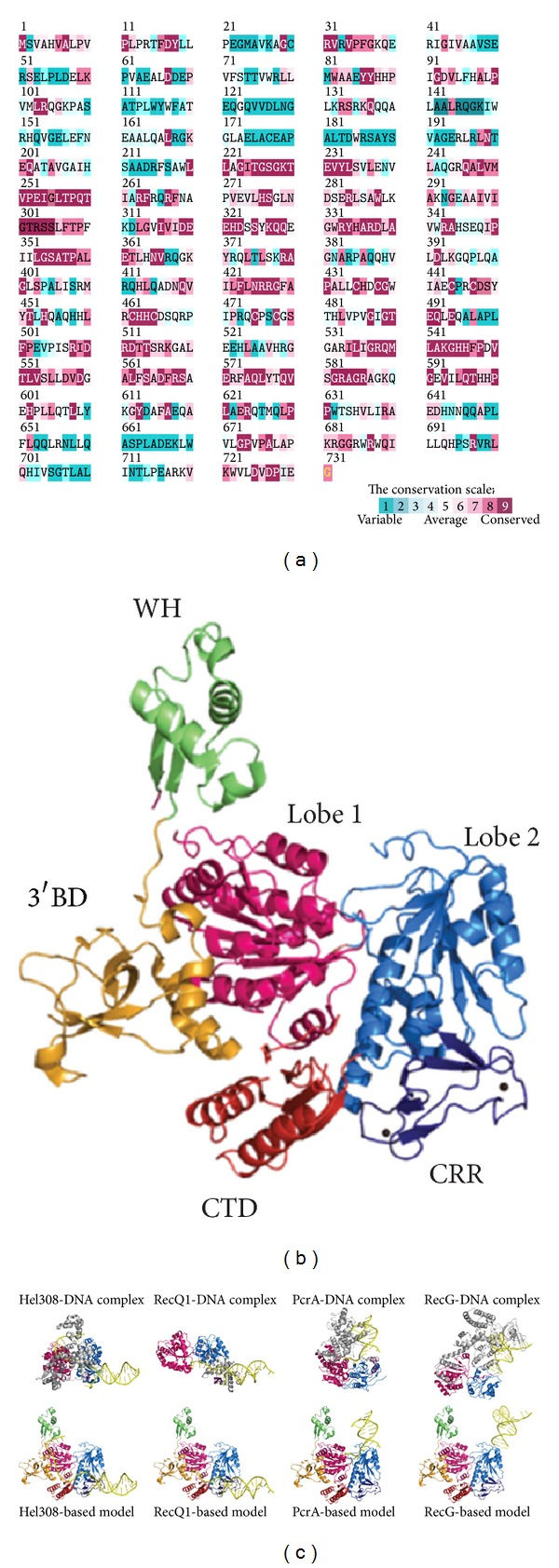
(a) Amino acid sequence alignment of KpPriA. An alignment consensus of 150 sequenced PriA homologs by the program ConSurf reveals the degree of variability at each position along the primary sequence. Highly variable amino acids are colored teal, whereas those highly conserved are colored burgundy. A consensus sequence was established by determining the most commonly found amino acid residue at each position relative to the primary sequence of KpPriA. The N-terminal 19–219 amino acid residues in PriA are not highly conserved. Asp17, located in the 3′BD of EcPriA, is crucial for the 3′ base-non-selective recognition of DNA, and Arg697, located in the CTD of KpPriA, is crucial for the SSB-Ct binding and induction of structural changes in the SSB-DNA complex; both are significantly invariable. (b) Crystal structure of KpPriA. KpPriA has six subdomains (Protein Data Bank entry: 4NL4), namely, a 3′ DNA-binding domain (3′BD; orange), a winged-helix domain (WH; green), two lobes of the helicase core (colored hot pink and blue, resp.), a Cys-rich region (CRR; dark blue), and a C-terminal domain (CTD; red). 3′BD and WH comprise the N-terminal DNA-binding domain (DBD), and the other four subdomains (two lobes of the helicase core, CRR, and CTD) comprise the helicase domain (HD). (c) Putative DNA-binding mode of KpPriA. The DNA-binding models of KpPriA are directly constructed by manually superimposing the KpPriA with DNA-bound crystal structure of Hel308 (Protein Data Bank entry: 2P6R), RecQ1 (Protein Data Bank entry: 2WWY), PcrA (Protein Data Bank entry: 3PJR), and RecG (Protein Data Bank entry: 1GM5). Considering the known ssDNA-binding site at DBD and the putative wedge element in KpPriA located at CRR, KpPriA may use the Hel308-based model to bind DNA. The *β*-hairpin, an important motif for DNA strand separation by helicase, is colored in magenta.

Figure 3(a) shows that the alignment consensus of 150 sequenced PriA homologs by ConSurf [44] reveals the degree of variability at each position along the primary sequence. The highly variable amino acid residues are colored teal, whereas the highly conserved are colored burgundy. A consensus sequence was established by determining the most commonly found amino acid residue at each position relative to the primary sequence of* K. pneumoniae* PriA (KpPriA). The amino acid sequences of KpPriA and EcPriA share 88% identity [45]. The N-terminal 19–219 amino acid residues in PriA are not highly conserved. The crystal structure of KpPriA has been recently determined [45]. KpPriA has six subdomains (Figure 3(b)), namely, a 3′ DNA-binding domain (3′BD; orange), a winged-helix domain (WH; green), two lobes of the helicase core (colored hot pink and blue, resp.), a Cys-rich region (CRR; dark blue), and a C-terminal domain (CTD; red). The 3′BD and WH comprise the N-terminal DNA-binding domain (DBD), and the other four subdomains (two lobes of the helicase core, CRR, and CTD) comprise the HD. Asp17, located in the 3′BD of EcPriA, is crucial for the 3′ base-non-selective recognition of DNA [43], and Arg697, located in the CTD of KpPriA, is crucial for the C-terminal tail of SSB (SSB-Ct) binding and induction of structural changes in the SSB-DNA complex [45]; both are significantly invariable. Many biochemical and genetic studies have been performed on the DNA-binding mode of PriA [7, 8], but the structural basis for the full length PriA-DNA complex is still lacking.

To elucidate the structural mechanism of DNA binding and unwinding by PriA, Bhattacharyya et al. [45] compared the structure of the full length KpPriA with those of other DNA helicases of superfamily II, namely, RecQ1 (Protein Data Bank entry: 2WWY) [46, 47] and Hel308 (Protein Data Bank entry: 2P6R) [48]. The structures of these helicases have been solved in complex with substrate DNA. RecQ1 and Hel308 bind to single-stranded DNA tailed duplex and unwind via the DNA unwinding wedge element, a prominent *β*-hairpin for strand separation [47, 49]. PriA also shares sequence similarity with other helicases, such as PcrA (Protein Data Bank entry: 3PJR) [50], a DNA helicase of superfamily I, and RecG (Protein Data Bank entry: 1GM5) [51], a DNA helicase of superfamily II. The structures of these helicases bound to DNA, along with KpPriA, are shown in Figure 3(c) for comparison. According to the crystal structures of the helicase-DNA complex, the two lobes of the helicase core of KpPriA (colored hot pink and blue, resp.) are aligned and manually superimpose the location of the dsDNA from the complex structure with KpPriA structure. These modeled structures of KpPriA show that the DNA-binding modes and thus the DNA-unwinding modes are different. Considering the known ssDNA-binding site at DBD and the putative wedge element in KpPriA located at CRR, KpPriA may use the Hel308-based model to bind DNA. The DNA-binding mode, fork DNA recognition site(s), and the helicase translocation using either the inchworm stepping or Brownian motor mechanism [52] must be further confirmed by additional biophysical and structural studies.

### 2.2. PriB Protein

PriB is a basic accessory protein in PriA-directed DNA replication restart primosome [11, 13]. It was originally discovered as an essential factor for the conversion of single-stranded circular DNA to the replicative-form DNA of *ϕ*X174 single-stranded phage in vitro. In contrast to the *ϕ*X174 model,* del(priB)302* mutant has almost wild-type phenotypes [53], suggesting that PriB is not absolutely required for bacterial DNA replication. PriB was formerly known as the “n protein” because it can be inactivated by treatment with* N*-ethylmaleimide [54]. In a PriA-PriB-DnaT-dependent reaction, PriB is the second protein to be assembled in the protein-DNA complex. It stabilizes the binding of PriA to DNA hairpin [35, 55] and then stimulates PriA helicase activity [40, 56]. The PriA stimulation by PriB correlates with the ability of PriB to form a stable PriA-PriB-DNA complex [40]. PriB also facilitates the association of DnaT with PriA [57]. More than one PriA-PriB complex is possibly involved in the initiation of primosome formation, and the effect of PriB on the PriA-DNA association is dependent on the DNA structure [58]. PriB interacts with PriA [15, 39], DnaT [15, 59, 60], SSB [54, 61], and itself [61, 62] and does not interact with DnaA, DnaB, DnaC, or DnaG [61]. The mechanisms of DnaC-DnaB complex loading by PriA-PriB-DnaT complex at the forks and then DnaB-DnaG complex formation remain unclear.

**Figure 4:**
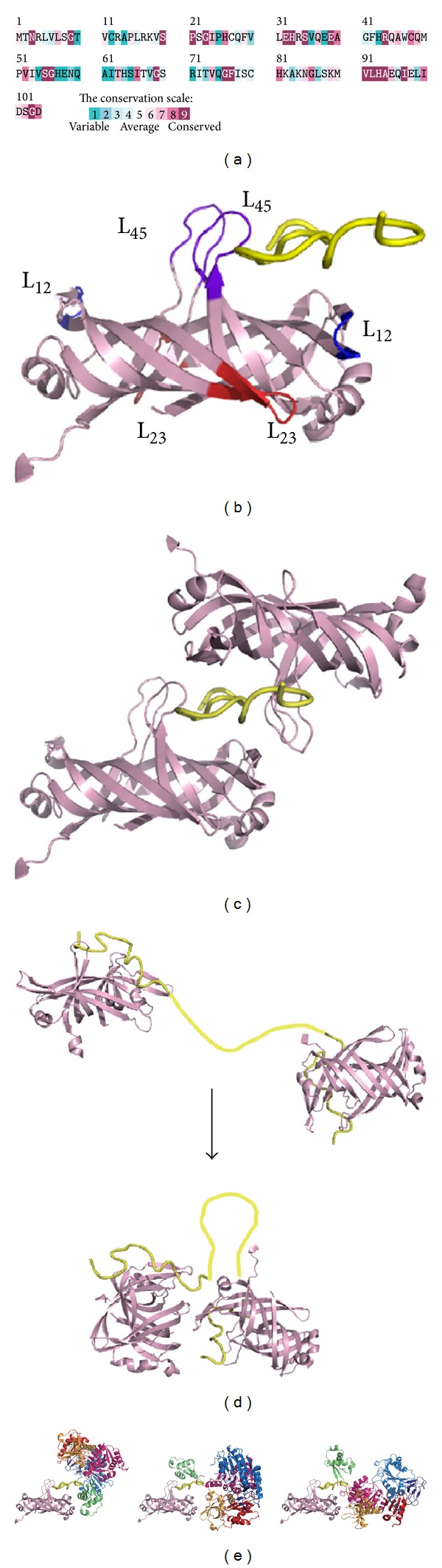
(a) Amino acid sequence alignment of EcPriB. An alignment consensus of 111 sequenced PriB homologs by the program ConSurf reveals the degree of variability at each position along the primary sequence. In general, the overall amino acid sequences among PriB proteins are not highly conserved, including many residues found important for ssDNA binding by EcPriB, such as Phe42, Trp47, Lys82, Lys84, and Lys89. (b) EcPriB is a homodimer with polypeptide chains of 104 amino acid residues. Each PriB monomer has an OB-fold structure with three flexible *β*-hairpin loops: L_12_ (residues 20–24; colored in blue), L_23_ (residues 37–44; colored in red), and L_45_ (residues 81–88; colored in purple blue). The ssDNA in the complex is shown in gold. (c) Crystal structure of EcPriB in complex with DNA. The complex structure of EcPriB (Protein Data Bank entry: 2CCZ) shows that a single dT15 ssDNA periodically interacts with two OB folds from two symmetrically related EcPriB dimers in the crystal and that the DNA is sandwiched by PriB dimers via their L_45_ loops. (d) Possible working model of interaction between two PriB proteins on ssDNA. PriB proteins cooperatively bind to ssDNA in two steps: two PriB proteins independently interact with ssDNA and then interact with each other through His64 on the ssDNA. The ssDNA in the complex is shown in gold. The region in ssDNA that does not directly interact with PriB, proposed in this two-step binding model, is colored in yellow. (e) Proposed models for PriA-DNA-PriB structure. These models are based on these observations: (1) two PriB dimers are complexed with a single dT15; (2) PriA has a highly electropositive ssDNA-binding region in DBD, and the basic DBD in PriA may be involved in complex with PriB; (3) DBD of PriA alone in solution forms a dimer and not a monomer as the full-length PriA.

PriB is a homodimer with polypeptide chains of 104 amino acid residues [63–65] (Figure 4(a)). Each PriB monomer has an oligonucleotide/oligosaccharide-binding (OB) fold structure [66–69] with three flexible *β*-hairpin loops: L_12_ (residues 20–24), L_23_ (residues 37–44), and L_45_ (residues 81–88) (Figure 4(b)). PriB can bind to ssDNA [15, 39, 40, 54, 56, 62–65, 70–73], ssRNA [65], double-stranded DNA [56, 70], and circular *ϕ*X viral DNA [73]. Although PriB is a dimer, it has only one DNA-binding site [73], which is located in loop L_45_ centrally within the dimer, and this site occupies a total site size of 12 ± 1 nucleotides [72]. The N-terminal 1–49 amino acid residue region of PriB is crucial for dimerization, whereas the C-terminal 50–104 amino acid residue region is crucial for ssDNA binding [71]. PriB shares structural similarity with the N-terminal DNA-binding domain of the* E. coli *SSB (EcSSB) [63–65, 74, 75]. Sequence comparisons and operon organization analyses also show that PriB evolves from the duplication of the SSB gene [76], but they differ in their ssDNA-binding properties and strategies [70, 73]. For example, EcSSB possesses three conserved aromatic residues (Trp40, Trp54, and Phe60) in the L_45_ loop of the OB fold. These residues serve important functions in ssDNA binding. Two of these residues (Trp40 and Phe60 in EcSSB) are replaced with nonconserved amino acids in the PriB family. In contrast to the EcSSB-DNA complex, the L_23_ loop from each subunit of PriB makes a close contact with the *β*-barrel core. The longer and extended L_23_ loops in EcSSB greatly increase the interactions between EcSSB and ssDNA [73, 75].

Figure 4(a) shows the alignment consensus of 111 sequenced PriB homologs by ConSurf [44]. The alignment indicates that the overall amino acid sequences among PriB proteins are not highly conserved; only 21 amino acid residues are significantly conserved: Asn3, Gly9, Ser20, Pro21, Gly23, Glu32, His33, Ser35, Glu39, Arg44, Ser55, Gly56, Gly69, Gly76, Phe77, Val91, Leu92, His93, Ala94, Ile97, and Gly103. Many residues important for ssDNA binding by* E. coli* PriB (EcPriB), such as Phe42 [64], Trp47 [64, 73], Lys82 [64, 73], Lys84 [73], and Lys89 [73], are not conserved. PriB may be a nonessential facilitating factor in DNA replication restart [53], and many prokaryotic genomes do not contain a recognizable homolog of* priB* [39]. Hence, we speculate that these residues among PriB proteins for binding ssDNA do not need to be precisely conserved.

We previously described the crystal structure of EcPriB in complex with ssDNA dT15 (Protein Data Bank entry: 2CCZ) [73]. A single dT15 ssDNA periodically interacts with two OB folds from two symmetrically related EcPriB dimers in the crystal, sandwiched by PriB dimers via their L_45_ loops (Figure 4(c)). Although the precise function of more than one PriB self-assembled on DNA to form a high-density nucleoprotein complex is still unclear, PriB binds DNA with strong cooperativity [70, 72, 73] in two steps (Figure 4(d)): two PriB proteins independently interact with ssDNA in primary binding mode, and then the proteins interact with each other through His64 on the ssDNA [77]. Whether the resultant ssDNA bound by more than one PriB forms a unique structure suitable for further assembly process for the primosome is not clearly known. The complex structure [73] and the thermodynamic analysis [72] indicate that the PriB dimer behaves like a protein with half-site reactivity, where only one monomer of the PriB dimer can engage in interactions with the DNA and the partner protein. The importance of the binding site on PriB for ssDNA to overlap the binding sites for PriA and DnaT needs to be investigated [15]. Each preprimosome may contain two PriB dimers [60]; whether or not this cocrystal structure, in which two PriB dimers are complexed with a single dT15 ssDNA, is an artificial or an actual binding mode for ssDNA by PriB also remains unclear. PriA may have a function similar to a monomer of the symmetrical PriB dimer in the crystal to stabilize the partially disordered ssDNA because the cooperation between PriB and PriA may be necessary to form a stable PriA-DNA-PriB complex. That is, the PriB-ssDNA-PriB complex (Figure 4(c)) may mimic the structure of the PriA-ssDNA-PriB complex (Figure 4(e)). We proposed three binding ways by use of the crystal structures of PriA and PriB. EcPriA has a highly electropositive ssDNA-binding region (amino acid residues 1–198) containing 8 Lys and 14 Arg residues in DBD; thus, the basic DBD in EcPriA may be involved in complex with EcPriB [73]. The DBD of EcPriA alone in solution forms a dimer and not a monomer as EcPriA [31], suggesting that another unknown stabilization factor is needed. The DBD of PriA and one of the monomers of PriB may bind to ssDNA cooperatively to decrease the dissociation rate of PriA from the DNA during helix unwinding [73]. The crystal structure of PriA in complex with PriB and DNA is necessary to elucidate the assembly mechanism of the replication restart primosome.

**Figure 5:**
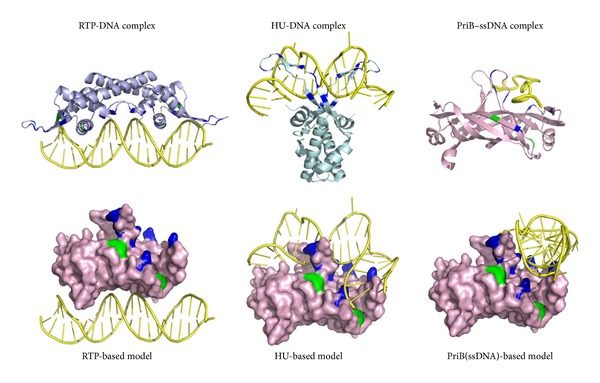
Putative dsDNA-binding mode of PriB. The DNA-binding models of PriB are directly constructed by manually superimposing the PriB dimer with DNA-bound crystal structure of RTP (Protein Data Bank entry: 1F4K), HU (Protein Data Bank entry: 1P51), and B-form dsDNA. The hydrophobic (green) and basic residues (blue) of RTP, Lys14, Arg16, Lys51, Arg59, Lys71, Lys74, Lys76, Lys77, Lys81, Lys91, Tyr58, and Tyr88, located on the dsDNA-binding surface, are indicated. The basic residues Arg53, Arg55, Lys56, Arg58, Arg61, Lys64, Lys68, and Arg75 of HU located on the dsDNA-binding surface are also indicated. Considering the known dsDNA-binding sites in PriB, PriB may use the HU-based model to bind dsDNA. Alternatively, PriB may use a similar approach to bind ssDNA and dsDNA because the residues responsible for ssDNA and dsDNA binding are almost overlapped.

More than a mere ssDNA-binding protein, PriB can bind both ssDNA and dsDNA with comparable affinity [70]. SSB can also bind dsDNA but with far less affinity than ssDNA [78]. According to the crystal structures of some dimeric proteins complexed with dsDNA found in the Protein Data Bank, PriB binds dsDNA in three possible ways (Figure 5). First, PriB may bind to dsDNA via the replication terminator protein- (RTP-) binding mode (Protein Data Bank entry: 1F4K) [79]. RTP, a dimeric WH protein [80, 81], uses two recognition helices to bind the major grooves of dsDNA. The PriB dimer also has two helices but does not contain any aromatic or positively charged residues as RTP. Thus, PriB binds to dsDNA via the RTP-binding mode that can be ruled out. Second, PriB may bind to dsDNA via the HU-binding mode (Protein Data Bank entry: 1P51) [82, 83]. HU is a dimeric nucleoid-associated protein that mainly uses two *β* sheets to bind dsDNA. Third, PriB may bind dsDNA in a manner similar to binding ssDNA. The structure-based mutational analysis indicates that the residues in PriB crucial for ssDNA binding are also crucial for dsDNA binding [70]. These residues responsible for ssDNA and dsDNA binding are almost overlapped; thus, PriB may use a similar approach to bind to the phosphate backbone of ssDNA and dsDNA through several positively charged residues. This phenomenon may be the reason for the comparable binding affinities of PriB to ssDNA and dsDNA. We speculate that, during evolution [76], the conserved aromatic and other residues in the L_45_ loop of the OB fold in SSB are changed into nonconserved and positively charged residues in PriB to more precisely fit the requirement for assembly of the replication restart primosome at the stalled DNA forks.

### 2.3. DnaT Protein

DnaT is an essential protein in the assembly of the PriA-directed DNA replication restart primosome [6, 11–13, 15, 55, 57]. It provides a specific recognition site for loading the replicative DnaB helicase during the promosome assembly [15, 42]. DnaT, formerly known as the “protein i” [84–86], was originally discovered as a critical factor for the conversion of single-stranded circular DNA to the replicative-form DNA of *ϕ*X174 single-stranded phage [9] and pBR322 plasmid replication, but not for R1 plasmid replication [87]. Genetic analysis for* E. coli* DnaT suggests that a replication protein is essential for bacterial cell growth because the colony size, cell morphology, inability to properly partition nucleoids, UV sensitivity, and basal SOS expression of the* dnaT822* mutant are similar to those of* priA2::kan* mutants [18]. DnaT is required for* E. coli* growth at elevated pressure [88] and for the lytic cycle of Mu growth [89]. DnaT is a homotrimer of ~22 kDa subunits [86, 90], but it also exists in solution as a monomer-trimer equilibrium system [91]. In a PriA-PriB-DnaT-dependent reaction, DnaT is the third protein to be assembled in the protein-DNA complex (Figure 2). The association of DnaT with PriA is facilitated by PriB [57]. Although the function of DnaT in the recruitment of DnaB helicase has been proposed, the fundamental function of DnaT for the replication restart primosome assembly is not widely known.

**Figure 6:**
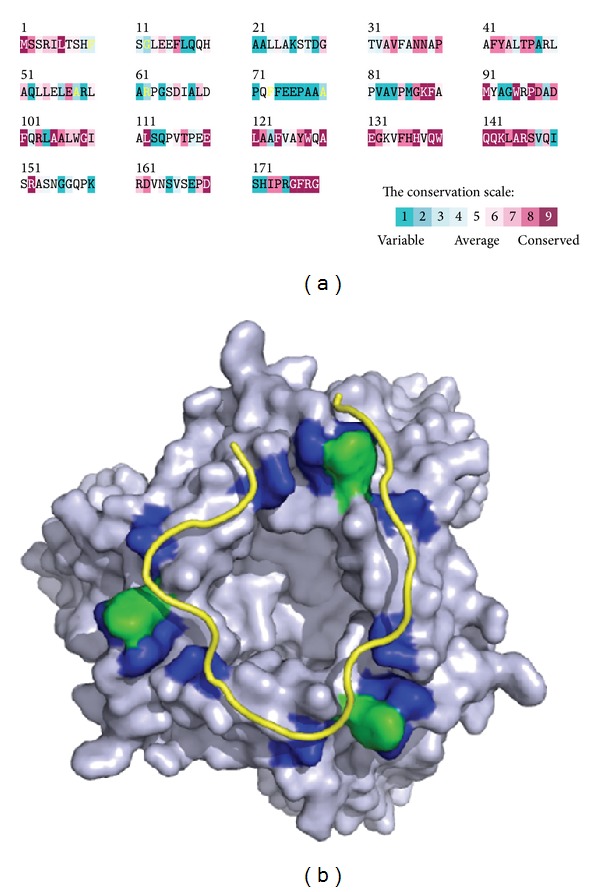
(a) Amino acid sequence alignment of KpDnaT. An alignment consensus of 29 sequenced DnaT homologs by the program ConSurf reveals the degree of variability at each position along the primary sequence. In general, the amino acid residues in the C-terminal region of KpDnaT are highly conserved. (b) Modeled structure of KpDnaT. The structure of KpDnaT is modeled by the bioinformatic program (PS)^2^ and then manually built using threefold symmetry with a 25 mer ssDNA (gold). The highly conserved hydrophobic (green) and basic residues (blue) of KpDnaT, His136, His137, Trp140, Lys143, Arg146, and Arg151 located on the potential ssDNA-binding surface are indicated.

We have recently identified and characterized that DnaT is a ssDNA-binding protein [90]. Based on the alignment consensus of 29 sequenced DnaT homologs by ConSurf [44], we found that the amino acid residues in the C-terminal region of* K. pneumoniae* DnaT (KpDnaT) are highly conserved (Figure 6(a)) and that KpDnaT contains 10 Arg, 5 Lys, and 18 aromatic amino acid residues (11 Phe, 4 Trp, and 3 Tyr). We attempted to assess whether or not KpDnaT, especially at the C-terminal region, has ssDNA-binding activity because the aromatic stacking and electropositive interactions serve important functions in ssDNA binding by proteins [73, 75, 92–94]. KpDnaT can form distinct complexes with ssDNA of different lengths, and the size of the binding site is 26 ± 2 nucleotides for a trimeric KpDnaT [90]. Although DnaT is not an OB-fold protein predicted from sequence analysis and structure modeling, the activity for ssDNA binding by DnaT, assayed in the same manner, is even higher than that of PriB, an OB-fold protein [90]. The two-domain structure for DnaT is characterized by the involvement of the N-terminal domain (amino acid residues 1–83) in PriB binding and the C-terminal domain (amino acid residues 84–179) in ssDNA binding [59].

To date, little is known about the ssDNA-binding mode of non-OB-fold proteins, particularly trimeric proteins. No protein with amino acid sequence similar to DnaT is found in the structure databank. Thus, homology modeling for the DnaT structure by several homology-based programs is not successful, including the use of SWISS-MODEL (http://swissmodel.expasy.org/) [95]. To obtain an indepth understanding of the structure-function relationship of KpDnaT, its 3D structure has been modeled by the bioinformatic program (PS)^2^ [96, 97]. (PS)^2^ (http://140.113.239.111/~ps2v2/docs.php) is an automatic homology modeling server that combines both sequence and secondary structure information to detect the homologous proteins with remote similarity and target-template alignment [96, 97]. The modeled structure of KpDnaT, manually built using threefold symmetry with a hit of alpha-aminotransferase from* Pyrococcus horikoshii* (Protein Data Bank entry: 1GD9) suggested from (PS)^2^, is a ring-shaped trimer (Figure 6(b)) [59, 90]. Based on the structural model of KpDnaT, we suggested that the positively charged (blue) and aromatic residues (green) located in the C-terminus of DnaT are involved in ssDNA binding: H136, H137, W140, K143, R146, and R151 (Figure 6(b)). These residues in DnaT are significantly conserved among the 29 sequenced DnaT proteins (Figure 6(a)). F73 and F74 are also potential binding sites for ssDNA, but they are not completely conserved in DnaT family. The ring-like structure of KpDnaT is slightly similar to that of the hexameric (comprised of three dimers) DnaC helicase from* Geobacillus kaustophilus*, a DnaB-like helicase [92]. DnaT may bind to DnaB with a stoichiometry of 1 : 2, one DnaT monomer to a DnaB dimer. However, the DnaT structure is only a modeled structure, and these speculations, including the putative DNA and DnaB-binding modes of DnaT, must be further confirmed by additional biophysical studies.

## 3. Perspectives

Most DNA helicases of superfamily I and superfamily II are almost nonhexameric and have poor dsDNA unwinding activities when acting alone in vitro [98]. Some helicases might function as ssDNA translocases rather than helicases, and self-assembly and/or interactions with accessory proteins are required to activate helicase activity [98]. Several monomeric ssDNA translocases of superfamily I can potentially displace proteins that are bound to ssDNA by translocating along the ssDNA and be activated by self-assembly, removal of an autoinhibitory domain, or direct interactions with an accessory protein(s) [38, 40, 99–101]. For PriA, the self-assembly and removal of an autoinhibitory domain for higher helicase activity have not been reported. However, poor helicase activity for PriA, which can be significantly stimulated by PriB [40] and SSB [38], is found. Based on the structure of KpPriA bound to an SSB C-terminal peptide (Trp-Met-Asp-Phe-Asp-Asp-Asp-Ile-Pro-Phe) and the study of a single-molecule FRET (smFRET), Bhattacharyya et al. [45] proposed a pushing mechanism, which is similar to that for the RecA recombinase [102], for PriA-mediated replication restart. For SSB-bound DNA replication forks, PriA translocase activity may push SSB along the lagging-strand template to expose additional ssDNA for PriB and DnaT binding and that will ultimately serve as a binding site for DnaB [45]. This model provides structural insight into the molecular mechanism for initiating replication restart primosome assembly. The interaction of PriB with PriA is weak, and the stimulation of PriA by PriB via an interaction with ssDNA is not DNA structure-specific [40]. Thus, the targeting of stalled forks and recombination intermediates during replication restart likely correlates with PriA alone. More structural studies for these primosomal proteins are still necessary to elucidate the interaction between PriB and DnaT, as well as the release from the replication restart system. Several studies have raised new interesting questions as to whether or not PriA, PriB, and DnaT are always synchronically expressed for physiological needs and whether or not PriB and DnaT have additional functions for other systems. PriA and DnaT are required for* E. coli* growth at elevated pressure [88]; however, why PriB is not necessary to be synchronically expressed has yet to be determined. Many prokaryotic genomes do not contain a recognizable homolog of* priB* and* dnaT* (e.g.,* P. aeruginosa*; Table 2). Thus, further operon and gene regulation analyses for PriB and DnaT expression, not limited to replication restart, should be also investigated in combination with the biochemical and structural investigations.

## Figures and Tables

**Table 1:** List of the structures of the primosomal proteins available in Protein Data Bank.

	PDB ID	X-ray	NMR	Length
PriA	2D7E	The N-terminal domain of *Escherichia coli* PriA		105
	2DwN	The N-terminal domain of *Escherichia coli* PriA bound to AG		105
	2D7G	The N-terminal domain of *Escherichia coli* PriA bound to AA		105
	2D7H	The N-terminal domain of *Escherichia coli* PriA bound to CCC		105
	2Dwl	The N-terminal domain of *Escherichia coli* PriA bound to AC		105
	2Dwm	The N-terminal domain of *Escherichia coli* PriA bound to AT		105
	4NL4	*Klebsiella pneumoniae* PriA bound to ADP		731
	4NL8	*Klebsiella pneumoniae* PriA bound to SSB C-terminal tail peptide		731

PriB	2CCZ	*Escherichia coli* PriB bound to ssDNA (15 mer)		104
	1V1Q	*Escherichia coli* PriB		104
	1WOC	*Escherichia coli* PriB		100
	1TXY	*Escherichia coli *PriB		100
	2PNH	*Escherichia coli* PriB E39A		100
	4APV	*Klebsiella pneumoniae* PriB		102
	3K8A	*Neisseria gonorrhoeae* PriB		100
	4FDB	*Ralstonia solanacearum* PriB		99
	3EN2	*Ralstonia solanacearum* PriB		95
	3FHW	*Bordetella parapertussis* PriB		102
	3KLW	*Bordetella pertussis* PriB		98
	4GS3	The N-terminal domain of *Thermoanaerobacter tengcongensis* PriB		104

DnaT		None		

DnaB	4ESV	*Geobacillus stearothermophilus* DnaB bound to DNA (14 mer)		441
	2R6E	*Geobacillus stearothermophilus* DnaB		441
	2R6D	*Geobacillus stearothermophilus* DnaB		441
	2R6A	*Geobacillus stearothermophilus* DnaB bound to DnaG		441
	2R6C	*Geobacillus stearothermophilus* DnaB bound to DnaG		441
	4M4W	*Geobacillus stearothermophilus* DnaB bound to DnaG and DnaI		454
	2R5U	The N-terminal domain of *Mycobacterium tuberculosis* DnaB		167
	2Q6T	*Thermus aquaticus* DnaB		440
	3GXV	The N-terminal domain of *Helicobacter pylori* DnaB		121
	4A1F	The C-terminal domain of *Helicobacter pylori* DnaB		323
	4NMN	*Aquifex aeolicus* DnaB bound to ADP		434
	2VYF	*Geobacillus kaustophilus* DnaC		441
	2VYE	*Geobacillus kaustophilus* DnaC bound to ssDNA (9 mer)		441
	1B79	The N-terminal domain of *Escherichia coli* DnaB		128
	1JWE		The N-terminal domain of *Escherichia coli* DnaB	114

DnaC	3EC2	*Aquifex aeolicus* DnaC 42–221	The N-terminal domain of *Bacillus subtilis* DnaI	180
	3ECC	*Aquifex aeolicus* DnaC bound to ADP		185
	2W58	*Geobacillus kaustophilus* DnaI		199
	4M4W	*Geobacillus stearothermophilus* DnaB bound to DnaG and DnaI		278
	2QGZ	*Streptococcus pyogenes* DnaI		308
	2K7R			106

DnaG	3B39	*Escherichia coli* DnaG 109–427 bound to ssDNA (15 mer)		321
	1DD9	*Escherichia coli *DnaG 115–428		338
	1DDE	*Escherichia coli *DnaG 115–428		338
	1T3W	The C-terminal domain of *Escherichia coli* DnaG		148
	2HAJ		*Escherichia coli* DnaG 447–581	135
	4E2K	*Staphylococcus aureus* DnaG 108–428		321
	4EDG	*Staphylococcus aureus* DnaG 108–428 bound to ATP		321
	4EDK	*Staphylococcus aureus* DnaG 108–428 bound to GTP		319
	4EDT	*Staphylococcus aureus* DnaG 108–428 bound to ppGpp		321
	4EDV	*Staphylococcus aureus* DnaG 108–428 bound to ppGpp		321
	4EE1	*Staphylococcus aureus* DnaG 108–428 bound to CTP		321
	4EDR	*Staphylococcus aureus* DnaG 108–428 bound to UTP		321
	2LZN		*Staphylococcus aureus* DnaG 462-605	143
	1Z8S		*Bacillus stearothermophilus* DnaG 452–597	146
			
	4EHS	The C-terminal domain of *Helicobacter pylori* DnaG 438–559		122
	4M4W	*Geobacillus stearothermophilus* DnaB bound to DnaI and DnaG		143
	2R6A	*Geobacillus stearothermophilus* DnaB bound to DnaG		143
	2R6C	*Geobacillus stearothermophilus* DnaB bound to DnaG		143
	2AU3	*Aquifex aeolicus* DnaG 1–403		403

PriC	2RT6		The N-terminal domain of *Escherichia coli* PriC	98

Length and amino acid residues.

**Table 2:** Examples for some different PriA-directed primosome systems.

	PriA size (amino acid residues)	Partner proteins found in NCBI
*Escherichia coli* and *Klebsiella pneumoniae *	731	PriB, PriC, DnaT, DnaC, DnaB helicase, and DnaG
*Staphylococcus aureus *	802	DnaD, DnaB, DnaI, DnaC helicase, and DnaG
*Pseudomonas aeruginosa *	739	Only DnaB helicase and DnaG are found

PriA is conserved in bacteria, but its primosomal partners are not.
